# Will science save us?

**DOI:** 10.1016/j.ebiom.2020.102778

**Published:** 2020-04-27

As this Editorial goes to press, normal life for millions of people around the globe has ground to a surreal halt. We shelter in place–shuttering our businesses, cancelling social gatherings big and small, and minimising all but the most essential of human interactions to slow the spread of a novel and deadly human respiratory virus, SARS-CoV2. This virus is the causative agent of COVID-19 and was first identified a few short months ago in December 2019. Although psychologically challenging and economically devastating, physical distancing is thought to be our best hope to minimise casualties, help protect our frontline caregivers by reducing the surge in numbers of hospital cases, and buy some time to formulate a counterattack.

And counterattack we most surely will. Laboratories and clinicians around the world have banded together, working against the clock to assess and subdue our common tiny enemy. Together, we seek therapies to treat the sick and vaccines to protect the susceptible. We seek a scientific solution.

Until a vaccine is available, one of our best shots at fighting COVID-19 in the clinic might be to repurpose drugs that have been tested in humans for other diseases. Remdesivir and hydroxychloroquine are two such repurposed drugs being tested in multiple clinical trials around the world. Remdesivir is a nucleotide analogue that was originally studied as a treatment for Ebola and Marburg viruses, but has been shown to be effective at inhibiting viral replication of several coronaviruses including the novel SARS-CoV2 in the laboratory. Trials of remdesivir in China and the USA are due to report initial findings in the coming weeks, and two phase 3 trials have been announced in the UK on April 2. The malaria drug hydroxychloroquine has also received increased attention after a small randomised controlled trial (RCT) from China was posted on March 31, before peer-review, on the preprint server, medRxiv. This unpublished study was limited to a small cohort of mildly or moderately ill patients but suggested that hydroxychloroquine could shorten time to clinical recovery and might help to clear pneumonia. However, another study published on March 30 in the journal *Medecine et Maladies Infectieuses* showed that patients with severe infection did not see a similar therapeutic benefit when given hydroxychloroquine in combination with the antibiotic azithromycin. Larger RCTs are needed to determine not only whether hydroxychloroquine is effective against COVID-19, but also whether the severity of disease and other factors might play a role in treatment outcome.

A subset of patients with severe COVID-19 disease might succumb to a hyper-inflammatory response known as cytokine storm, in which an overactive immune system can wreak havoc on the lungs and other parts of the body leading, in worst cases, to multi-organ failure and death. Clinical trials are also underway to test whether drugs that target the proinflammatory cytokine interleukin (IL)-6, such as toxilizumab or sarilumab, could be an effective treatment in this subset of patients. However, given its intended purpose of resolving infection, blocking the inflammatory response might not be without risks. The timing of treatment could be an important consideration for any approach aimed at manipulating inflammation.

On March 24, Mount Sinai Hospital in New York (New York, USA) announced that they would begin testing plasmapheresis of convalescent serum on patients who have COVID-19 who are critically ill. The US Food and Drug Administration has approved this treatment for compassionate use in patients with severe cases of disease and other hospitals in the USA and globally are launching similar treatment protocols. The method has been used for more than 100 years and is based on a simple concept: isolate virus-specific antibodies from the blood of recovered COVID-19 patients, and infuse these into infected patients to neutralise virus, providing protective immunity. There are some indications from a small case series from China published in *JAMA Network* on March 27 that broader studies might be warranted. In this preliminary study, five severely ill patients were treated with donor plasma containing IgG and IgM anti SARS-CoV-19 antibodies, and all five patients showed signs of improvement after 1 week of transfusion. This case series has some important limitations that should be noted, including absence of untreated controls and the fact that concurrent treatments might have played a role in the outcome.

Never before have scientists and clinicians united with such scale and singular focus. Based on a small number of preliminary studies, a large coordinated effort is now underway to properly vet our best available therapeutic weapons against COVID-19. We wait for the research to tell us if these therapies are safe and effective enough to help us through this phase of the battle. All the while, behind the scenes, we work on a long-term strategy against the virus.

*EBioMedicine*

